# Rurality and relative poverty drive acquisition of a stable and diverse gut microbiome in early childhood in a non-industrialized setting

**DOI:** 10.21203/rs.3.rs-5361957/v1

**Published:** 2024-12-02

**Authors:** Victor Seco-Hidalgo, Adam Witney, Martha E Chico, Maritza Vaca, Andrea Arevalo, Alexander J Schuyler, Thomas AE Platts-Mills, Irina Chis Ster, Philip J Cooper

**Affiliations:** St George’s University of London; St George’s University of London; Fundación Ecuatoriana Para la Investigación en Salud; Fundación Ecuatoriana Para la Investigación en Salud; Fundación Ecuatoriana Para la Investigación en Salud; University of Virginia; University of Virginia; St George’s University of London; St George’s University of London

**Keywords:** rural, poverty, gut microbiome, environment, tropics

## Abstract

There are limited longitudinal data from non-industrialized settings on patterns and determinants of gut bacterial microbiota development in early childhood. We analysed epidemiological data and stool samples collected from 60 children followed from early infancy to 5 years of age in a rural tropical district in coastal Ecuador. Data were collected longitudinally on a wide variety of individual, maternal, and household exposures. Extracted DNA from stool samples were analyzed for bacterial microbiota using 16S rRNA gene sequencing. Both alpha and beta diversity indices suggested stable profiles towards 5 years of age. Greater alpha diversity and lower beta diversity were associated with factors typical of rural poverty including low household incomes, overcrowding, and greater agricultural and animal exposures, but not with birth mode or antibiotic exposures. Consumption of unpasteurized milk was consistently associated with greater alpha diversity indices. Infants living in a non-industrialized setting in conditions of greater poverty and typically rural exposures appeared to acquire more rapidly a stable and diverse gut bacterial microbiome during childhood.

## Introduction

The human gut microbiome develops rapidly during early infancy and during the transition from a diet of breastmilk to solid foods, becoming stable by 3 to 5 years of life^1–6^. The conservation of a healthy gut microbiome is considered to play an important role in the maintenance of health over the life course^7–10^. The gut microbiota fulfills several important functions, initially in the development of the immune system, and later in immune and metabolic homeostasis^7, 11–15^. Alterations or imbalances of the gut microbiome, known as dysbiosis, have been linked to a wide variety of chronic non-communicable diseases (NCDs) such as obesity, diabetes, cardiovascular and liver diseases, colorectal cancer, neurological disorders, inflammatory bowel disease, and allergic disorders^16–23, 3, 15^.

Host and environmental factors considered to determine the development and composition of gut microbiota have been extensively studied in high-income countries^24, 25, 3, 9, 26^. Factors considered to mold the developing gut microbiome include those in the child’s postnatal environment, particularly contacts with the microbiome of maternal mucosal and epithelial surfaces^27^, the household and external environment in which the child is raised, breastfeeding and post-weaning diet, antibiotic exposure, and presence of household pets or older siblings^1, 28, 8, 3, 29, 30, 27, 6, 26, 31^.

Because dysbiosis of the gut microbiota may have a role in the mediation of disease, it is important to understand how gut microbiota develops during childhood and the factors that determine this process. There is conflicting evidence on the role of alterations in gut microbiota composition in the development of NCDs^1, 28, 23, 3, 30, 26^. Potential sources of inconsistency between studies include not just differences between populations, geographic locations, and environmental living conditions, but also differences in study design, and analytical approaches.

Most evidence for a role of microbiota in disease mediation is derived from cross-sectional studies that are unable to distinguish the temporal sequence between dysbiosis and disease. Most longitudinal studies of the postnatal development of the gut microbiome and its determinants have been from high-income settings^32, 33^. There are still relatively few such longitudinal studies from resource-poor non-industrialized settings in low and middle-income countries (LMICs)^34–36^, particularly in Latin America.

LMIC populations are presently undergoing demographical, epidemiological, and nutritional transitions^37, 38^ that have accompanied the shift from traditional to modern lifestyles, and have, as a consequence, become increasingly vulnerable to the development of NCDs^39^. The determinants of gut microbiome development in non-industrialized settings may differ from those in high-income countries (HICs) because of marked differences in living conditions, lifestyle and socio-cultural factors, as well as the presence of enteric pathogens such as soil-transmitted helminths (STH). Further, longitudinal studies in such settings provide the opportunity to study the effects of factors that cannot be studied in more affuent settings because they are either absent (e.g STH parasites) or ubiquitous (e.g. clean water and sanitation).

In the present analysis, we used a birth cohort from a rural tropical region of coastal Ecuador to study longitudinally the development of gut microbiota composition from early infancy to 5 years of age and to explore the effects on microbiota development of a wide variety of maternal, individual, household, other environmental exposures including antibiotics and STH parasites.

## Results

### Characteristics of study sample

A total of 238 stool samples from 60 children (56.7% boys) with a median of 4.5 samples per child at different ages (range 1–7), were analyzed between birth to 5 years of age. The distributions of child, maternal, socio-economic, and household characteristics are shown in Table 1. Most children were delivered vaginally (78.3%) with a mean birthweight of 3.3 kg (SD 0.6). During infancy, mean length of exclusive breastfeeding was 4.1 months (SD 2.2; median [Q1-Q3], 5.5 [3–6]) and weaning on to a family-type diet started at mean of 6.2 months (SD 1.81; median 6 [5–7]). During the first 5 years of life, 91.7% of children had consumed unpasteurized milk (56.7% consumed it frequently), 13.3% had attended daycare, 55.0% had received antibiotics, 37.7% had an STH infection (*A. lumbricoides* 31.7% vs. *T. trichiura* 36.7%), and 86.7% had been treated with anthelmintic drugs. In terms of maternal characteristics, 23.3% were illiterate, 20.0% were of Afro-Ecuadorian ethnicity, almost half (48.3%) had an STH infection during the child’s gestation, and half (50.0%) had taken at least one course of antibiotics during the pregnancy. The households to which these children belonged were poor: 90% earned less than the equivalent of 1 basic monthly salary of US$480 (median income US$170, range 80–700). Half (50.0%) of households were rural, 58.3% were overcrowded (i.e >=3 persons/sleeping room), 50.8% were constructed with traditional materials (i.e wood and bamboo), 25% had access to potable water, 15% had access to a sewage system (via a water closet), 38.3% had farm animals around the house (any of pigs, cows, and horses), and 48.3% had at least one household member with an STH infection.

### Gut microbiota composition

Reads from only 1 of the 238 stool samples were discarded (i.e. <10,000 reads). Of 237 remaining samples, a total of 28,033,042 high-quality reads (median 109,163 reads/sample) were mapped to 33,448 unique OTUs classified into 18 phyla and 273 genera (one bacterial group was unclassified at both levels). Read counts from two non-template controls were 1 each and not considered further. An exploratory NMDS Bray-Curtis-derived scatter plot for distributions of samples by age illustrated the effects of age on beta diversity (Figure S1).

### Microbiota diversity indices and associations with potential explanatory variables

**Figure 1:**
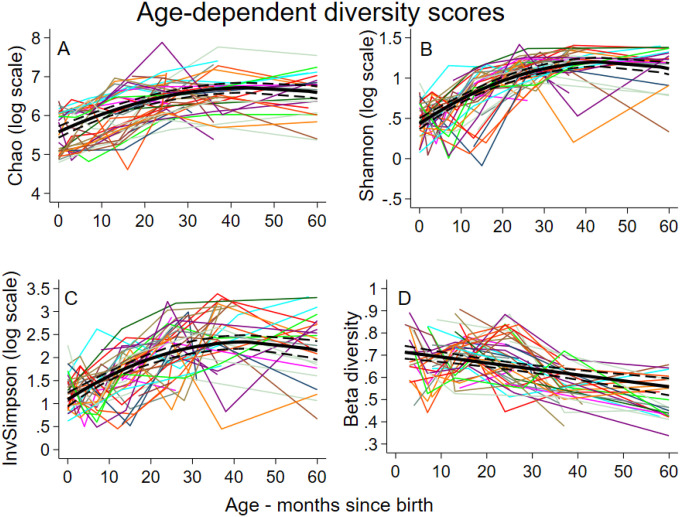
Age-dependent scores for the main indices of alpha diversity (Chao, Shannon, and InvSimpson) and age-dependent changes in beta diversity of bacterial microbiota in fecal samples from 60 children.

Average log-transformed values for intestinal alpha diversity indices (i.e., Chao, Shannon, and InvSimpson) showed nonlinear associations with age (up to 4-degree polynomial patterns) (P<0.001) (Figure 1A–C). The average values for alpha diversity indices rose rapidly during infancy to peak at 45 (95% CI 41–49), 45 (95% CI 42–48), and 43 (95% CI 39–48) months for Chao, InvSimpson, and Shannon, respectively (Table S1), after which trajectories tended to stabilize. Beta diversity declined in a linear fashion (Figure 1D) indicating that monthly rate of change in microbiota across the cohort declined with age.

Age-adjusted associations between individual, maternal, and household characteristics and alpha and beta diversity measures are shown in Table 1 and Figs S2-S5. Chao diversity, an index weighted towards measuring OTU richness, was significantly positively associated with exclusive breastfeeding duration (GMR 1.05, 95% CI 1.00–1.10), a diet richer in traditional foods (GMR 1.37, 95% CI 1.09–1.70), frequent consumption of unpasteurized milk during childhood (frequent vs. never, GMR 1.23, 95% CI 1.01–1.49), household overcrowding during childhood (GMR 1.30, 95% CI 1.01–1.67), lower socioeconomic status (higher vs. lower GMR 0.67, 95% CI 0.51–0.87) at birth, use of traditional materials for household construction at birth (non-traditional vs. traditional, GMR 0.73, 95% CI 0.56–0.95), and having a peri-domiciliary horse during childhood (GMR 1.34, 95% CI 1.09–1.64). The effects of house construction materials, overcrowding, socio-economic status - all markers of poverty as well as the presence of dogs in the house interacted with age such that the effects of these factors on diversity were greater in early infancy but attenuate with increasing age. Shannon diversity (a metric for both evenness and richness but which places greater weight on richness) was significantly positively associated with frequent consumption of unpasteurized milk during childhood (frequent vs. never, GMR 1.16, 95% CI 1.04–1.30) and agricultural exposure (GMR 1.10, 95% CI 1.01–1.20) but inversely associated with maternal (GMR0.93, 95% CI 0.86–1.00) and household STH infections (GMR=0.90, 95% CI 0.83–0.96). There was evidence that Shannon diversity became greater in children higher in the birth order after 2 years of age (interaction with age P value=0.039). Inverse Simpson diversity (an index that provides inference about community evenness and richness, but which places greater weight on evenness) increased markedly among children consuming unpasteurized milk during childhood compared to those with no reported consumption, an effect which emerged during the first 6 months of life. There was some evidence that Inverse Simpson scores increased with having peri-domestic cows (GMR 3.42, 95% CI 1.50–7.73), an effect which emerged after the first 6 months and dissipated in late childhood (Interaction P=0.041); but was lower in households with STH infections (GMR 0.81, 95% CI 0.68–0.97).

Beta diversity is a measure of the distance in microbial composition between different samples that may be from the same or different individuals. In this analysis, beta diversity refers to changes between samples with age in which a fecal sample is compared to the previous chronological sample from the same individual (i.e. representing ordered temporal observations). The resulting two-level hierarchical dataset allowed the estimation of the average monthly rate of change in diversity that declined with age (Figure 1D). Estimates were adjusted for the age at which the first stool was collected (median (Q1-Q3) =1.5 (0–24) months [i.e., 75% of the children had their first sample prior to 24 months]). Figure 1D shows predictions starting at around 3 months of age. There were age-interactions with relative affluence of households (interaction P=0.007), maternal education (interaction P=0.004), and household STH infections (interaction P=0.013) on beta diversity such that diversity seemed to stabilize more rapidly among children with illiterate mothers, those living in poorer households, and those with household members not infected with STH, despite opposite effects during early childhood. There was some evidence for an impact of dietary patterns on beta diversity: greater ingestion of vegetables and fats (p=0.014) and sweets (p=0.022) produced opposite effects with low levels of vegetables and fats and high levels of sweets showing greater changes in diversity.

A summary of exposure-specific associations with alpha and beta diversity is provided in Table 3.

### Relative abundance at Phylum level

**Figure 2 Age:**
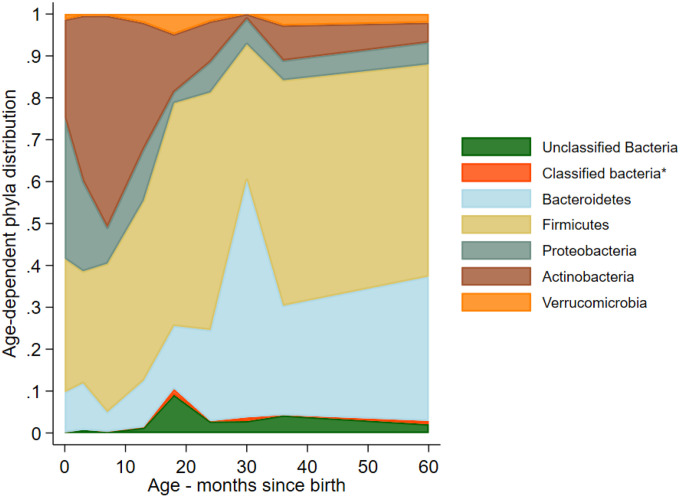
-dependent distributions of bacteria OTUs identified at phylum levels in fecal samples from 60 children. *Classified bacteria group included Chlamydiae, Cyanobacteria_Chloroplast, Deinococcus_Thermus, Elusimicrobia, Fusobacteria, Lentisphaerae, Planctomycetes, Spirochaetes, Synergistetes, TM7 and Tenericutes.

Most bacterial OTUs identified in the feces of cohort children corresponded to the phyla, *Bacteroidetes, Firmicutes, Proteobacteria, Actinobacteria* and *Verrucomicrobia* (Figure 2). There was a shift in phyla dominance from *Proteobacteria* and *Actinobacteria* in early infancy to *Bacteroidetes* and *Firmicutes* in later childhood*: Bacteroidetes* and *Firmicutes* increased in relative abundance from approximately 40% in early infancy to 80% by 5 years, while *Proteobacteria* and *Actinobacteria* decreased from 60% to less than 20% over the same period.

### Ratio of Prevotella and Bacteriodes genera

**Figure 3:**
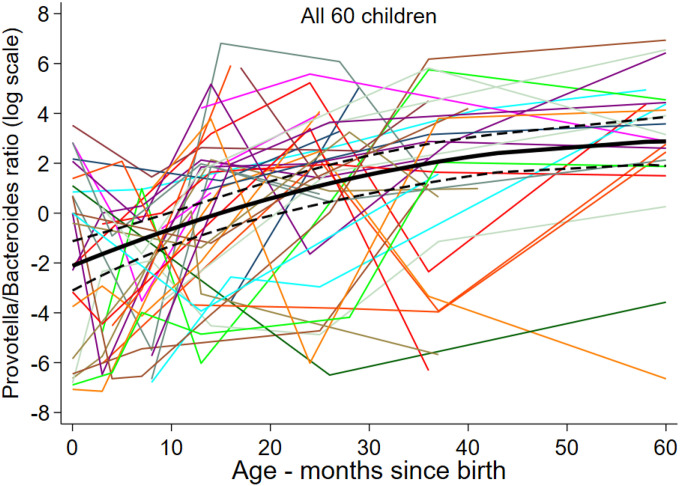
Age-dependent ratio of *Prevotella* to *Bacteriodetes* (log scale).

The *Prevotella/Bacteroides* ratio increased steadily with age reaching a maximum at 5 years of age (Figure 3). Factors positively associated with the *Prevotella/Bacteroides* ratio (Table 2 and Figure S6) were: STH infections during childhood (factor=3.96, P=0.033); maternal STH infections (factor=4.22, P=0.011); no maternal antibiotics during pregnancy, an effect that was lost with increasing age (interaction P= 0.011); lower socio-economic status (higher vs. lower, factor=0.076) in early but not later childhood (P=0.007); fewer material goods (more vs. less, factor=0.24), having a latrine (WC vs, latrine, factor=0.27, P=0.038), agricultural exposures at birth (factor=8.97, P=0.005) and during childhood. In the case of agricultural exposures, the effect was lost in later childhood (birth effect, interaction P=0.036; during childhood effect, interaction P=0.015). There was some limited evidence for a positive association with rural versus urban residence (factor=3.21, P=0.052).

## Discussion

There are limited longitudinal data from non-industrialized settings in LMICs on the development of the gut microbiome during early childhood^40, 35, 36^ and the broader role of the child’s living environment in guiding this development. To our knowledge, this is the first such study from Latin America^41^. We used data from a population-based birth cohort to study the variability in and development of the bacterial gut microbiome during the first 5 years of life among healthy children from a marginalized population in a rural district of tropical coastal Ecuador. We investigated the potential effects of a wide variety of individual, maternal, and household factors on the developing bacterial microbiome to identify potential exposures, or patterns of exposures, that might have a role in determining microbiota development in such a setting. Exposures were measured either at birth or repeatedly during childhood and had one of two possible effects on diversity trajectories – either they shifted the trajectory curve up (or down) with similar rates of change from early infancy through childhood at different exposure levels or rates of change at the different levels were different resulting in a loss or even reversal of the effect. Our analytic strategy allowed us to address overall effects of these exposures on longitudinal changes in the gut microbiome over time rather than during specific developmental time windows.

Several studies from HIC settings have indicated that the maturation profile of the early infant microbiome starts with colonisation by a limited number of taxa during the first months after birth and then expands to reach a stable microbiome community after 3–5 years^42, 1–6, 33^. Here, we observed rapid increases in alpha diversity metrics during early infancy that peaked and stabilized after 3 years of age. In contrast, declines in rates of change in beta diversity were relatively constant during childhood.

Diet is a major driver of development of the gut microbiome, initially through breastfeeding in infancy and later at the time of weaning with the introduction of the family diet^43^. Diet affects the gut microbiome both as source of exogenous bacteria that can seed the intestine^44^, and by the nutrient environment it provides within the intestine that favors the survival of bacteria able to process available nutrients. Here, a greater period of breastfeeding was associated with greater bacterial richness from early infancy, an effect that appeared to be maintained during childhood. Previous studies have shown variable effects of breastfeeding on gut bacterial diversity during infancy – breastfeeding was associated with lower gut bacterial diversity in early infancy^45^, an effect that appeared to be lost by 6 months^46, 47^.

Weaning is associated with the acquisition of a greater bacterial load and diversity and is a key driver of gut microbiome maturation^42, 48^. Weaning likely explains the shift in phyla dominance from *Proteobacteria* and *Actinobacteria* in early infancy to *Bacteroidetes* and *Firmicutes* in later childhood, observed here as for previous studies done in HIC settings^32, 33, 49, 50^. *Bacteroidetes* and *Firmicutes* may allow more efficient breakdown of complex carbohydrates^48^. The greater consumption of unpasteurized milk was the only factor measured in this study that was consistently associated with increases in alpha diversity indices and marginally also with a decreased decline in beta diversity. Although unpasteurized milk is considered to confer beneficial effects, for example in protecting against childhood atopy and asthma^51, 52^, to our knowledge, there is only one published study of the effects of unpasteurized milk on the human gut microbiome – a study of the effects over a 12-week period of adult volunteers – which showed increases in the relative abundance of *Lactobacillus*^53^.

There was some evidence also that consumption of a ‘traditional’ diet (in this setting representing a diet rich in cereals, tubers and starches, legumes, and seafood) was associated with increased microbial richness and is consistent with previous findings of greater gut microbial diversity being associated with ‘traditional’ diets in a variety of settings including Nigeria^54^, Mexico^55^, Japan^56^, the Atlantic region of Southern Europe^57^, the Mediterranean, and in Nordic countries^58^. Traditional diets, which tend to be rich in complex carbohydrates, are considered beneficial to health through their effects as pre- and probiotics, and increased bioavailability of short-chain fatty acids (SFCAs) and antioxidants^55^. A diet rich in processed sugars (the ‘sweets’ dietary pattern) did not appear to affect trajectories of alpha diversity indices but was associated with an upwards shift in beta diversity trajectories while a plant-based diet had the opposite effect. Greater consumption of processed sugars has been associated with reduced gut bacterial diversity^59–61^.

Factors associated with greater poverty, particularly rural poverty, seemed to be associated with greater gut bacterial diversity. Children living in overcrowded and less affluent households seemed to have a richer microbiota from early infancy as did those with household dogs and exposures to peri-domestic large animals. Trajectories for alpha diversity indices more indicative of evenness were shifted upwards in children with a greater number of older siblings and more frequent agricultural exposures including to peri-domestic cows. Some exposure effects (e.g. breastfeeding and unpasteurized milk) persisted into later childhood while others disappeared (e.g. overcrowding and the sibling and large animal effects) or even reversed (i.e. household dogs). Previous studies have shown associations of increased microbial diversity with household dogs^62, 63^, siblings^62, 48, 2, 3, 64^, and farm exposures^65–67^. These effects likely relate to the acquisition of a more diverse bacterial microbiome from environmental sources with which the child has close contacts^68^, and may represent surrogate markers for soil and other outdoor green space exposures. This is the first study to address the longitudinal effects of the acquisition of STH infections during childhood on gut microbiota and showed, unexpectedly, that these STH infections might reduce diversity. Previous systematic reviews of studies of the effects of STH on the gut microbiome in children, largely from cross-sectional studies, have provided evidence for greater alpha diversity measures in infected compared to uninfected children^69, 70^. To our knowledge, there are no previous analyses of the effects of the early childhood acquisition of STH on developmental trajectories of the gut bacterial microbiome. Our data do not support a major role for STH in determining developmental trajectories of the gut microbiome in early childhood.

Monthly declines in beta diversity during childhood indicated a reduction in the distance between sequential samples (from the same individual) with increasing age and likely reflects the gradual acquisition of a more stable and presumably adult-like gut microbiota. This is consistent with observations that the variation in microbial composition between children decreases with increasing age^3, 33^. This monthly rate of decline in beta diversity observed here was affected by dietary factors - diets rich in vegetable and fats and those low in sweets were associated with an increased rate of decline. These within-individual changes in beta diversity were minimal among children acquiring STH and those living in the least affluent households and those with illiterate mothers, perhaps indicating the rapid acquisition of a more stable microbiota even from early infancy in these children. This poverty effect might be explained partly by an accelerated maturation of the gut bacterial microbiota in early infancy, and before collection of initial samples against which subsequent changes were compared.

Particularly of interest in this study were the factors that were not significantly associated with biodiversity indices, and which have been identified as being important in previous studies. Among such factors were delivery mode and antibiotic use during pregnancy and childhood. Previous studies have shown strong effects of both on the infant gut microbiome^4, 5, 71, 3, 2, 72^. The birth mode effect may become attenuated over time^73^. Antibiotics are of interest because of the disruption they cause to gut microbiota composition, particularly during the establishment of the microbiome in early life^4, 5, 71^, although the evidence for long-term effects of antibiotics during infancy on gut microbiota is inconsistent^73^. It has been suggested that the disruption of gut microbiota by antibiotics during early childhood may have profound consequences for health later in life^74^. Our data appear to indicate that both caesarean section and antibiotic treatments during childhood have limited long-term effects on longitudinal trajectories of the diversity indices measured but do not discount important effects during critical developmental time windows such as during the first 3 months of life. The lack of effect of these exposures in our study setting might be explained by rapid colonisation (at birth) or recolonisation (after antibiotics) from family members and a living environment rich in sources of these bacteria.

Studies comparing the gut microbiota of adults living in rural traditional settings in Africa and Latin America compared to US and European cities have emphasized the importance of *Prevotella* and *Bacteriodes*, belonging to the Phylum Firmicutes, as core taxa that distinguish gut microbiota between these very different settings^75, 1, 76, 77^. The ratio of OTUs representing these two genera, has been used as an indicator for a more traditional versus modern lifestyle with a higher ratio indicating less modernization (or ‘Westernization’)^75, 1, 76, 77^. It has been suggested that the observed geographic differences in this ratio may be driven by differences in dietary patterns from a more diverse traditional diet enriched in plant-derived carbohydrates compared to a diet high in animal protein and fats, sugars, and starch^78^. A study of South-East Asian migrants to the US showed a shift to greater *Bacteriodes* (vs. *Prevotella*) with increasing period of residence that was associated with a loss in the capacity to degrade dietary fibre^79^. In this study we were able to collect detailed dietary information on study children, although at a later age, and our data did not show an association with dietary patterns. Rather, the ratio of *Prevotella* to *Bacteriodes*, that increased overall in this cohort during childhood, was associated with factors more typical of rural poverty, namely STH infections, agricultural exposures, and household poverty (i.e., no bathroom and fewer material goods). Interestingly, maternal antibiotics during pregnancy appeared to have the reverse effect during early infancy. An STH effect in favoring *Prevotella* has been observed previously^69^.

The age-adjusted incidence of chronic non-communicable diseases is increasing in LMICs^80^, particularly in marginalized and transitional populations^81, 82^. Numerous factors are considered to have contributed to this trend including urbanization processes that are transforming the living environments, social and economic relations, and lifestyles of rural populations^83, 84^. An important effect of urbanization has been to reduce the biodiversity of microbial communities resulting in the acquisition of a depleted gut microbiome during early childhood. This has been labeled ‘microbiota insufficiency syndrome’ and while likely to be most marked in the ‘industrialized’ microbiota of non-affluent families living in HICs^85^, may also be emerging during the process of urbanization in LMICs. The long-term consequences of a depleted microbiota may include increased vulnerability to chronic diseases associated with impaired immune and metabolic homeostasis^85–87^ including chronic respiratory and cardiometabolic diseases. Our data, from a population of healthy children living in a transitional setting in coastal Ecuador undergoing rapid changes relating to urbanization, have provided novel insights into the factors that mold gut bacterial microbiota development during childhood and will allow us to explore, in future analyses, how developmental trajectories of gut microbiota might affect the later regulation of inflammatory and metabolic responses and disease development.

## Strengths and limitations

Important strengths were the longitudinal nature of the study, the relatively long period of follow-up (from early infancy to 5 years of age), the unusual setting (compared to most previous longitudinal studies) in a rural marginalized LMIC population, and the wide range of exposures measured that included individual and environmental factors including STH infections. The effects on diversity of several exposures such as childhood STH infections and agricultural were evaluated during infancy rather than at a single point in time likely improving the validity of exposure measurements. The analytical strategy used all available observations while accounting for their inherent hierarchical structure, unlike commonly used paired tests that are valid only if the number of observations are the same on each occasion. This analysis was exploratory given important limitations. Our sampling and analytic strategies only allowed us to address the long-term longitudinal effects of patterns of exposures on microbiome development trajectories rather on specific critical time windows of development or the changes in the specific composition of the microbiome at high resolution. A major limitation was the relatively small sample size with limited study power for measuring effects of multiple exposures. Many of the exposure-microbiome associations observed may be subject to confounding, particularly for strongly correlated exposures linked to rural poverty including agricultural exposures. However, any attempt to control for confounding for such strongly associated exposures would be di cult to interpret because of residual confounding. More important, however, are overall patterns linked to groups of correlated exposures. Further, we had limited power for microbiome effects occurring before 3 months given the limited number of samples collected before this time point. The bacterial 16S rRNA gene sequencing method used here employed standardized methodology from the Earth Microbiome project^88^ but which yields relatively short reads compared to more recent technologies. These short reads provide basic data on diversity and taxonomic composition of bacterial communities, but not to species or strain level, were sufficient to meet the objectives of the present study. Data on dietary patterns were collected when the same children were 6 to 8 years of age – children in this setting tend to be introduced to the family diet at weaning with relatively little modification, and as such, the data collected broadly represent the family diet and likely reflects that received by the child post-weaning.

## Conclusion

In the present study we used data from a birth cohort to study developmental trajectories of key parameters of the gut microbiome from early infancy to 5 years and explore their potential epidemiological determinants. The cohort was recruited in a non-industrialized setting in a rural district in a tropical region of coastal Ecuador. We took a more holistic approach in considering a wide variety of childhood exposures that might affect the developing gut microbiome. Our data indicate that children, living in conditions of poverty, particularly rural poverty in less affluent and overcrowded households with greater farming and animal exposures, tended to have longitudinal trajectories of greater bacterial diversity and acquired more rapidly a stable gut microbiome. Some exposure effects were strongest in infancy while others persisted during childhood. Consumption of unpasteurized milk was consistently associated with greater bacterial while STH infection risk or strongly correlated environmental exposures tended to reduce alpha diversity measures, particularly evenness. Delivery mode and antibiotic exposures did not appear to affect these developmental trajectories and indicate that such ‘unhygienic’ but biodiverse living environments in early childhood may rapidly compensate for any deficiencies or perturbations caused by such factors.

## Materials and methods

### Study design and sample selection

We analyzed fecal samples from a subsample of children in the ECUAVIDA birth cohort^89^. The ECUAVIDA cohort was a population-based birth cohort of 2,404 newborns whose families lived in the rural district of Quinindé, Esmeraldas Province, and were recruited around the time of birth at the Hospital Padre Alberto Buffoni (HPAB) in the town of Quinindé between November 2005 and December 2009. This population-based cohort was designed to study the effects of early life infections on the development of allergy and allergic diseases in childhood. The selection of the individuals in the study subsample was done based on rural or urban residence using administrative/geographic criteria, such that 50% of children included in this analysis lived in a rural location. The district of Quinindé is largely agricultural where the main economic activities relate to the cultivation of African palm oil and cocoa. The climate is humid tropical with temperatures generally ranging 23–32°C with yearly rainfall of around 2000–3000mm. Inclusion criteria were being a healthy baby, collection of a maternal stool sample, and planned family residence in the district for at least 3 years.

### Follow-up and sampling of children

Children were followed-up from birth to 8 years of age with data and stool samples collected at 1, 3, 7, 13, 18, 24, 30 months, and 3, 5, and 8 years of age. Follow-ups were done either by scheduled visits to a dedicated clinic at HPAB or by home visits. At the initial home visit, a questionnaire was administered to the child’s mother by a trained member of the study team to collect data on potential risk factors^89^. Maternal questionnaires were repeated at 7 and 13 months and 2, 3, 5, and 8 years of age. Questionnaires collected detailed information on individual and household factors including breastfeeding, diet, illness, farming, and animal exposures including household pets, and housing conditions. A food frequency questionnaire for the child’s dietary intake, developed and validated within the same study population, was administered to the child’s mother between 6 and 8 years of age^90^.

### Stool collection

Stools were collected into sterile plastic recipients from children during home and clinic visits. Stool samples were collected also from the mother during the last trimester of pregnancy or around the time of birth of the child and from all family members during a home visit done during the first two weeks of life of the child in the cohort. Stool samples were examined using four microscopic techniques to detect and/or quantify STH eggs and larvae including direct saline wet mounts, formol-ether concentration, modified Kato-Katz, and carbon coproculture^91^. All stool samples were examined using all 4 microscopic methods where stool quantity was adequate. A positive sample was defined by the presence of at least one egg or larva from any of the above detection methods. An aliquot of stool was preserved in 90% ethanol at −80°C for molecular analyses.

### DNA extraction, PCR, and sequencing

Whole genome DNA was extracted from 20 mg of stool using the FastPrep DNA for Soil Kit (MP Biomedicals Inc, Solon, OH, USA). The hypervariable region 4 (V4) of the bacterial 16S rRNA genes was amplified by PCR using the standardized protocols of the Earth Microbiome Project and yielding amplicons of 300–350 bp^88, 92, 93^. Paired-end sequencing reactions were performed on a MiSeq platform (Illumina, San Diego, CA, United States).

### Bioinformatics and statistical analyses

Sequencing data were debarcoded, paired-end overlapped and filtered using Mothur software, version 1.48.0^94, 95^ discarding long/short contigs, reads with homopolymers greater than 8 and chimeric sequences. Default parameters were used in picking Operational Taxonomic Units (OTUs) employing the SILVA database release 138.1^96^ as reference. Read counts were normalized to 10,000 sequences per sample measures. Alpha-diversity metrics (Chao, InvSimpson, and Shannon indexes) were derived using Mothur, while Beta diversity was assessed using quantitative Bray-Curtis distances for each pair of samples using the R package “vegan” (version 2.6–2). Chao, Shannon, InvSimpson indexes and Bray-Curtis distances were analyzed as longitudinal outcomes^97^ in conjunction with independent variables of which some were measured once at birth or later while others were time-varying. Statistical analyses were exploratory and estimated the age-adjusted effects of individual, household, and lifestyle factors on microbiota diversity measures and their changes with age (i.e. beta diversity). Analytical strategies were tailored to the longitudinal structure of the data and included random effects (mixed) models under assumptions of normality for outcomes. Alpha-diversity metrics were log-transformed and estimates for associations derived by back-transformation and interpreted using geometric mean ratios (GMR). Beta diversity, which considered age-adjacent individual pairs, estimated average monthly rates of change, adjusted for age using as starting baseline the first sample collected for each individual^98^. P-values less than 0.05 were considered statistically significant. Interactions of these factors with age were explored and presented when statistically significant. Mixed models for these continuous outcomes operated under missing at random assumption for missing observations which had a nonmonotonic pattern, while the analyses accounted for all available data points^99, 100^. Consumption patterns were derived *a priori* using principal components analysis as described^101^. The dietary patterns identified was guided by data interpretability^102^, internal consistency of the dimensions of the food frequency questionnaire was considered acceptable if Cronbach’s alpha > 0.65. Dietary patterns were identified as traditional (high in cereals, tubers and starches, legumes, and seafood), breakfast (high in bread/biscuits, fruit, sausages, milk and dairy products), sweets (high in sugars and sweet foods, snacks, coffee, and fizzy drinks), and plant-based (high in vegetables, legumes, vegetable oils and fats, and condiments), and categorized as ‘low vs. high’ using the median values as cut-offs from an analysis of 1,966 cohort children for whom data were available.

## Data Availability

The metadata used in this analysis are provided in Supplementary Materials. Sequence data have been submitted to the European Nucleotide Archive database with accession number PRJEB80800 (https://www.ebi.ac.uk/ena/browser/view/PRJEB80800).
